# Transversal analysis of public policies on user fees exemptions in six West African countries

**DOI:** 10.1186/1472-6963-12-409

**Published:** 2012-11-20

**Authors:** Valéry Ridde, Ludovic Queuille, Yamba Kafando, Émilie Robert

**Affiliations:** 1 Research Centre of the University of Montreal Hospital Centre (CRCHUM), Montreal, Canada; 2 Department of Social and Preventive Medicine, University of Montreal, Montreal, Canada; 3 Institut de recherche en sciences de la santé (IRSS) du CNRST, Ouagadougou, Burkina Faso

## Abstract

**Background:**

While more and more West African countries are implementing public user fees exemption policies, there is still little knowledge available on this topic. The long time required for scientific production, combined with the needs of decision-makers, led to the creation in 2010 of a project to support implementers in aggregating knowledge on their experiences. This article presents a transversal analysis of user fees exemption policies implemented in Benin, Burkina Faso, Mali, Niger, Togo and Senegal.

**Methods:**

This was a multiple case study with several embedded levels of analysis. The cases were public user fees exemption policies selected by the participants because of their instructive value. The data used in the countries were taken from documentary analysis, interviews and questionnaires. The transversal analysis was based on a framework for studying five implementation components and five actors’ attitudes usually encountered in these policies.

**Results:**

The analysis of the implementation components revealed: a majority of State financing; maintenance of centrally organized financing; a multiplicity of reimbursement methods; reimbursement delays and/or stock shortages; almost no implementation guides; a lack of support measures; communication plans that were rarely carried out, funded or renewed; health workers who were given general information but not details; poorly informed populations; almost no evaluation systems; ineffective and poorly funded coordination systems; low levels of community involvement; and incomplete referral-evacuation systems. With regard to actors’ attitudes, the analysis revealed: objectives that were appreciated by everyone; dissatisfaction with the implementation; specific tensions between healthcare providers and patients; overall satisfaction among patients, but still some problems; the perception that while the financial barrier has been removed, other barriers persist; occasionally a reorganization of practices, service rationing due to lack of reimbursement, and some overcharging or shifting of resources.

**Conclusions:**

This transversal analysis confirms the need to assign a great deal of importance to the implementation of user fees exemption policies once these decisions have been taken. It also highlights some practices that suggest avenues of future research.

## Background

In May 2011, the World Health Assembly (WHA 64.9) reminded all countries of the need to avoid point-of-service user fees in order to achieve universal coverage. This was in keeping with resolutions of the African Union and United Nations agencies favouring point-of-service user fees exemptions for pregnant women and children under the age of five years [1,2]. However, at the same time, this World Health Assembly resolution stressed the importance of promoting a sharing of experience among countries on this matter. This article is aimed at contributing to that exchange.

Faced with the financial barrier created by user fees generalized across Africa in the 1990s [3], more and more countries have chosen to implement user fees exemption policies. Most often these are aimed at certain services or categories of persons. Southern Africa was the first African region to embark on this initiative to find a solution to the population’s difficulties with financial access to services. Several surveys of the scientific literature on these recent policies have produced syntheses of the state of current knowledge [4-6], and in so doing have echoed the cautionary advice put forward when these policies were first implemented [7]. However, these surveys also show that in early 2009, when the project described in this article was conceived, scientific studies on these policies in West Africa were rare. At mid-July 2008, no scientific article on West Africa had yet been published [4] and by the end of 2009, to our knowledge, only one article on an experience in Niger had been published [8].

In fact, a series of cursory analyses done in 2009 of the implementation of these policies in several countries in the region clearly showed that the technicians putting these policies in place: i) often encountered the same operational challenges, sometimes made the same errors and did not borrow enough ideas from similar experiences; and ii) had experiences that, if put to use, would be helpful in producing pragmatic knowledge about these policies and drawing lessons from their implementation [9].

Indeed, just as researchers produce scientific knowledge, the experience of street-level bureaucrats [10] is a tacit source of knowledge that can be updated and put to use in a short time, without the long delays associated with producing scientific results [11-13].

Thus, the objective of this article is to present a transversal analysis of the results of a knowledge aggregation process undertaken with street-level bureaucrats regarding user fees exemption policies in six West African countries (Benin, Burkina Faso, Mali, Niger, Togo and Senegal). This analysis was based on the results produced by each of the six teams working in the countries during a knowledge aggregation exercise published in a report [14]. These case studies aimed to combine both tacit and scientific knowledge through a collaborative and participative process. Each team had performed a case study of one or more specific public policies, and we sought to understand whether, notwithstanding the heterogeneity of the studies, we could uncover any recurrent themes, points in common, as well as any innovations. Highlighting the elements that are convergent along with those that may be divergent is useful for drawing lessons on how policies were implemented in these six countries.

While all these policies were largely aimed at reducing the financial barrier to healthcare access in West Africa, they differed in terms of their contexts, the target publics and the services involved. In Table 1, some of the contextual indicators are compared.

**Table 1:** Some indicators from the six countries

	**Benin**	**Burkina**	**Mali**	**Niger**	**Senegal**	**Togo**
Population (in thousands, 2008)	8,662	15,234	12,706	14,704	12,211	6,459
Life expectancy at birth (years, 2008)	57	51	49	52	59	59
Neonatal mortality rate (per 1,000 live births, 2008)	33	36	52	34	34	33
< 5 years mortality rate (probability of death before the age of 5 years per 1,000 live births, 2008)	121	169	194	167	108	98
Maternal mortality ratio (per 100,000 live births, 2005)	840	700	970	1 800	980	510
Prenatal consultation rate (coverage by antenatal services (%): at least 1 visit in 2009)	84	85	70	46	87	84
Rate of assisted deliveries (births assisted by qualified health personnel (%), 2008)	78	54	49	18	52	62
Physician rate (per 10,000 inhabitants, 2009)	0.63	0.60	0.83	0.2	0.61	0.54
State spending on health (as a % of total State spending, 2007)	10.7	13.3	11.8	12.4	12.1	7.7
Total spending on health *per capita* in $ (PPA $ int., 2007)	70	72	67	35	99	68
Public spending on health per inhabitant (PPA $ int., 2007)	36	40	34	18	56	17
% GDP in health (total health spending as a % of gross domestic product, 2007)	4.8	6.1	5.7	5.3	5.7	6.1

Source: [15].

## Methods

The methodological approach used for this inter-country knowledge aggregation process was one of multiple case studies with several embedded levels of analysis [16].

The cases were made up of certain user fees exemption policies implemented in six countries. Case selection was instrumental to the process, in that it was based on feasibility and on the utility that country team members could draw from the results they would produce [17]. Table 2 summarizes the content of these policies, for which details are available elsewhere [14].

**Table 2:** Synthesis of the content of the policies studied

	**Benin**	**Burkina**	**Mali**	**Niger**	**Senegal**	**Togo**
**Services exempted (year of initiation)**	Caesareans (2009)	All types of deliveries (2006/2007)	Caesareans (2005) and antimalaria treatments (2007)	Caesareans (2005) and consultations for children under 5 years (2006)	Consultations for persons over the age of 60 years (2006)	ARV treatment for PLHIV (2008)
**Source of financing**	State	State	State (with partners for malaria inputs)	State and partners	State	State and partners
**Reimbursement method**	Fixed-rate reimbursement by the act	Fixed-rate reimbursement by the act (plus actual expenses)	Provision of inputs (and fixed-rate reimbursement of acts for caesareans)	Fixed-rate reimbursement	Drug vouchers and budget allocation	Provision of inputs
**Governance**	Independent national agency	Cell of the Ministry of Health’s Department of Health and Family	Steering committees	Free healthcare services steering committee of the Ministry of Health	Steering committee	National Anti-AIDS Program

Sources: country case studies and [18].

The teams carried out a framework analysis process [19] using a common framework to describe the context of the public policies studied and their content, as well their formulation, implementation and effects, thereby following the classic divisions of the study of public policies [12].

Each team used multiple sources of data to be able to triangulate the information. The persons in charge of country case studies received training sessions in methodology and were closely followed. Table 3 summarizes the methods used in each country. It should also be noted that, since the methodological approach adopted meant that the case studies were produced by people involved in the policies’ implementation, all the case studies thus benefited from data coming from the actors’ experiences, that is, participant observations used largely in internal team meetings (in each country) and regional groups (in Ouagadougou).

**Table 3:** Synthesis of the data sources used in each country

	**Benin**	**Burkina**	**Mali**	**Niger**	**Senegal**	**Togo**
**Documentation**	9 reports 7 decrees, memorandums, 2 theses	12 reports 1 memorandum 1 newspaper article 1 scientific article	15 reports 1 master’s thesis 3 laws, decrees and letters	12 reports 1 law 1 scientific article	17 reports 4 decrees or circulars 5 newspaper articles 1 thesis 1 scientific article	26 reports 5 decrees, laws and procedures
**Questionnaire**	-	-	-	-	30 persons	-
**Individual and group discussions**	15	-	-	10	33	25
**Country team workshops**	2	2	3	2	2	4

Sources: country case studies.

In Benin, the team consisted of a health sociologist, a public hospital manager and the director of an NGO supporting mutual health organizations. They conducted 15 individual interviews with managers in the Ministry of Health involved in these policies, did an in-depth documentary analysis, and held two team meetings.

In Burkina Faso, the team consisted of three Ministry of Health representatives from central departments and one district, two physicians, and a health attaché from an NGO supporting user fees exemption policies. This team carried out an in-depth documentary analysis and held two team meetings.

The Mali team consisted of five people from central departments of the Ministry of Health responsible for exemption policies and the medical coordinator of an NGO supporting these policies. The team carried out a large documentary study and conducted three workshops open to other people in the Ministry to obtain additional data.

In Niger, the team consisted of four people from departments of the Ministry of Health and three members of NGOs involved in supporting the implementation of user fees exemption policies. The team held two working sessions, conducted an in-depth documentary analysis, and conducted 10 qualitative interviews with members of the cell coordinating the policy, representatives of two NGOs, and persons from the Ministry of Health’s planning department.

In Senegal, the team consisted mainly of three people: a geriatrician in charge of a health facility, a consultant researcher in social policy, and a researcher in political science. The team used a self-administered questionnaire completed by 10 managers and five directors of healthcare facilities, as well as by 15 elderly persons. These respondents were randomly selected from health facilities in the Dakar region that were directly involved in implementing the free healthcare policy for elderly persons (i.e., Sesame plan). The team also organized three discussion groups, each with eight elderly persons, all of whom were randomly selected from among the patients of Dakar’s only geriatric centre. In addition, 25 qualitative interviews were carried out with five Sesame plan managers from the central level, two agents of the national health financing cell, 10 physician managers, and eight members of the health committees of the 10 health facilities that received the questionnaires. These interviews and discussions with a total of 49 people, conducted in French and in the national language, were recorded. Finally, a documentary analysis was also carried out.

In Togo, the team was made up of five managers of the national HIV program and two members of patients’ associations. The team carried out an in-depth documentary analysis, conducted 25 individual interviews of healthcare providers, persons living with HIV, and members of the Ministry of Health, and organized four team meetings, one of which was largely open to outside guests with useful information to document the policy.

To do the transversal analysis that synthesizes the knowledge produced by the countries, we focused our analytical approach on highlighting, on one hand, the policies’ main intervention components (the technical aspects of implementation), and on the other, the attitudes and reactions of the actors involved in these components. These implementation components and actor’s attitudes were identified by means of two exploratory workshops carried out with decision-makers in Burkina Faso and in Mali in June 2010 and a review of the available grey literature on exemption policies implemented in these six countries [18]. These two sources allowed us to uncover the existence of:

 • Five main implementation components: management (S1), communication (S2), monitoring / evaluation / coordination (S3), community involvement (S4), and patient management and referral (S5);

 • Five main actor’s attitudes : health workers’ motivation and satisfaction (A1), the provider–patient relationship (A2), patients’ satisfaction (A3), patients’ perceptions on health and on financial access to healthcare (A4), and health workers’ coping strategies (A5).

We thus synthesized the data produced by each country to highlight, first, the core elements from the perspective of this analytical framework [19], then the commonalities and some particular features. These latter features were specifically highlighted when they were innovative in relation to current policy processes implemented in West Africa [4,9]. Therefore, this transversal analysis was conducted based on three data sources.

The first source consisted of ongoing discussions and interactions between the authors of this article and the country team members regarding their specific case study. These discussions took place at three regional workshops between October 2010 and May 2011 that brought together all the authors of the country case studies, first to launch their studies, then to present progress reports. To prepare for the second and third workshops, the teams sent the case study document they were producing to the project team for systematic analysis. Then, at the general meetings, the case study authors presented their work verbally. The discussions and working groups that followed helped the country teams to continue working on their documents. The present transversal analysis also takes into account the content of these discussions.

The second source was the final versions of the case study documents, which are available elsewhere, see [14]. Taking into account the preliminary versions that each team produced, the country case studies were all read in their entirety several times, an essential process for thoroughly comprehending and analyzing their content.

The third source of data came out of the transversal analysis of the multiple case studies. In effect, at the final project workshop (May 2011), members of the country teams held several working group meetings in order to begin working on the transversal analysis by groups of three countries. A presentation of these three-country transversal analyses in a plenary session allowed us to pursue the analytical discussion further. In addition, the authors of this article presented to everyone in attendance their own preliminary transversal analysis based on the results of five countries. All of these discussions were captured using systematic note-taking and audio-recordings for subsequent analysis by the authors of the present article.

Except for Senegal, there was no need for ethical authorization since this was a process of self-reflection undertaken by people involved in the policies being studied. Their involvement was authorized by their respective institutions, essentially the ministries of health of their countries and the participating NGOs. The people interviewed were guaranteed anonymity upon providing informed consent to participation. In Senegal the Director of the gerontological public health facility in Dakar provided the ethical authorization to implement the study and to interview the patients.

## Results

The presentation of the transversal analysis is organized according to the two broad dimensions retained from the analytical framework, as well as their components. We will not revisit all the details of the implementation components and actors’ attitudes; only a few examples will be used to illustrate the analyses. Readers will find all the details in the country-specific case studies [14].

### Implementation components

#### ***Management (S1)***

This is the component that was covered in greatest detail by the authors of the knowledge aggregation exercises in the countries. This is undoubtedly explained by the fact that this was the core of their expertise and they were able to discuss the strengths and weaknesses of the processes under way.

##### A majority of State financing

One of the first lessons from this study is that exemption policies are essentially financed by the national budget. The support of funding agencies does not appear to be essential for implementing these policies, beyond the budget support that, of course, is a source of resources for the countries. This endogenous financing configuration pertains to exemption policies targeting categories of persons deemed vulnerable, such as the elderly in Senegal or pregnant women in Burkina Faso, Mali or Benin. However, when it comes to user fees exemptions for specific illnesses associated with the tradition of vertical programs, financing is generally exogenous. This is the case for anti-malarials in Mali or HIV drugs in Togo.

##### Maintenance of centrally organized financing

In all cases, financial management remained centralized, thereby maintaining the traditional functioning of regional administrations. Except for a certain portion of Burkina Faso, the administrative decentralization and skills transfer under way in all the countries have not yet been applied in these recent policies. In Niger, for example, the regional departments do not appear to play a role in financial control, and they are sometimes even bypassed in the transmission of reimbursement requests. There is one noteworthy exception. For reasons that are most certainly political, one local community in Senegal managed to free up, only once, 30 million F CFA to support the presidential decision. According to the chairman of the regional council, this decision was taken in order to maintain the commitment of the President of the Republic to the elderly and to show the regional council’s support for this population. This regional council chairman was an active member of the party in power of the President at that time [20]. In fact, the Sesame plan was a very political decision taken at a time when the incumbent President’s power was being contested, such that this local support for participation in funding this policy had electoral connotations [21].

##### Multiplicity of reimbursement methods

In contrast to the two preceding points, there does not seem to be any general trend regarding compensation mechanisms for health centres that exempt certain services from user fees. We therefore find at least four different ways of functioning: i) provision of inputs (ACT drugs and caesarean kits in Mali, ARVs in Togo, drug vouchers in Senegal); ii) fixed-rate reimbursement of acts (for children under five in Niger, deliveries in Burkina Faso until 2010, caesareans in Benin); iii) reimbursement of acts based on estimated actual expenses (deliveries in Burkina Faso since early 2011); iv) a mixed system (inputs for caesareans plus fixed-rate reimbursement of acts in Mali). No modality appears to predominate, which shows that there is no single solution and that solutions must necessarily be adapted to the implementation context. In the case of fixed-rate reimbursement, we have little information about the calculation methods, which sometimes appear to have over-assessed (Burkina Faso) or under-assessed (Niger) the costs. Finally, we should add that Senegal and Burkina Faso, for example, have sometimes organized their systems by pre-funding the anticipated acts, that is, by making a budget (fixed rates per act) available before the exemptions took effect in the health centres.

##### Reimbursement delays and/or stock shortages

All the countries, regardless of their selected financing method, have had to contend with bottlenecks in the reimbursement process. These difficulties might be temporary and without major consequences (Burkina Faso, Mali for caesareans), or they might jeopardize the policies (Niger and Senegal). In Mali, it is interesting to note that the shortages of caesarean kits experienced in the beginning stopped when the decision was taken to request that these be managed by a central department better equipped for this activity. In Senegal, the State owes two billion F CFA to hospitals alone, under the Sesame plan (which is in addition to the general debt of 12 billion F CFA, and without counting what is owed to health centres), while in Niger, the State owes more than 11 billion F CFA to health centres.

##### Almost no implementation guides

In most of the countries, policies have been implemented without any guides being produced to explain their content and functioning. Even when such guides did exist, as in Mali and Burkina Faso, they were rarely available to front-line health workers.

##### Lack of support measures

In every country alike, with the exception of Togo (HIV), the policies have not been adequately supported by measures to facilitate their implementation. It was as if the decision to abolish user fees, which in itself was difficult to achieve, was enough on its own. While there are only four geriatricians in Senegal, all in Dakar, additional training sessions were only organized starting in 2011. In Burkina Faso, no personnel was added to handle the additional administrative burden generated by the policy-specific accounting process, and it took years for the management software to become operational. In Niger, the software to manage the exemption was only installed in 2011.

#### Communication (S2)

##### Communication plans that were rarely carried out, funded or renewed

In all the countries, communication processes were largely evaded. Very little was done on this in Senegal, for example. Elsewhere, once the policies had been declared (sometimes relayed by the very few national media), few activities were carried out to ensure that all stakeholders were aware of the policies’ details. When such activities did occur, they were rarely funded to the level required (for example, 2% of the subsidy was planned for communication in Burkina Faso) and it was very difficult to obtain numerical data on this in the countries. Moreover, the budgets were not renewed. In other words, people who were not reached by the meagre information campaigns at the start of the policies did not receive any further attention later.

##### Health workers who were given general information but not details

Health workers, who were most affected by these policies that changed their practices, were relatively advantaged, as in most countries they appeared to know the general content of the exemption measures relatively well. However, in most cases, they did not know all the details, e.g. did the policies cover fuel for evacuations (Burkina Faso) or supplementary exams (Mali)?

##### Poorly informed populations

Several years after the policies went into effect, the populations’ knowledge of their provisions is far from perfect. It appears there are still people who are unaware of the existence of certain exemptions, as was shown, for instance, in one district of Niger. Moreover, as was the case for ARVs in Togo or deliveries in Burkina Faso, the complex and partial nature of the exemption measures made it difficult for the population to understand them. Thus, policies that are overly specific or exemptions that are incomplete sometimes complicate the information campaigns.

#### Monitoring / evaluation / coordination (S3)

##### Almost no evaluation systems

None of the countries appeared to have any rigorous and detailed evaluation plan. The data from the national health information systems (NHIS) were not used, or were not adapted, to analyze the policies’ effects. For example, in Mali there are no routine national statistics that make it possible to know the number of caesareans carried out *before* the policy. No country carried out any baseline population survey before introducing its policy, nor did the countries conduct any community surveys to verify whether the acts reimbursed to the health centres corresponded to the consultations actually carried out. In Niger, although external evaluations are normally required every three years and accounting audits every year, these have never been done. Overall, monitoring is perceived as an accounting and administrative function. However, for example, numerous changes to the accounting monitoring system in Burkina Faso have rendered it difficult to understand and use. Only Togo’s policy appears to have a useful monitoring/evaluation system, most certainly due to the involvement of persons with HIV, the extensive external funding, and its integration into the national anti-AIDS program, which has long experience with this evaluation exercise.

##### Ineffective and poorly funded coordination systems

Most of the countries set up a coordination cell specifically for this policy. Most often, these mechanisms were incorporated into the healthcare system, with the exception of Benin, which created an independent agency. However, this agency, which remains strongly politicized, replicated the same centralizing shortcomings of the public administration in its financial management, resulting in some delays in health centre reimbursements. Sometimes a decentralized level was set up for these mechanisms, like the focal points for user fees exemptions in Niger and Mali. Yet everywhere, these coordination systems were poorly organized and lacking in resources. In Senegal, the monitoring committee that was set up never met, and in Mali, there was no supervision of the policy in 2009 due to lack of funding. In Niger, the free healthcare services steering committee was created in 2010, five years after the implementation of the user fees exemption for caesareans and four years after the exemption for children under five.

#### Community involvement (S4)

In all the countries involved in this knowledge aggregation project, the policies were implemented in a context in which the Bamako Initiative (BI) had established community management committees in health centres.

##### Low levels of community involvement

In the countries in which user fees exemptions were organized, and where revenues were managed by management committees, these committees were often involved in receiving reimbursements. But this involvement remained very much limited to this accounting aspect for incoming funds or inputs. In addition, some committees experienced financial difficulties when they had to pre-finance certain free services when reimbursements were delayed. Community involvement rarely went beyond this accounting aspect. In Mali, this community involvement even deteriorated because the communities believed the policy would fund the referral-evacuation system and so they sometimes stopped their financial participation. On the other hand, the specificity of the policies in Togo (PLHIV) and Senegal (elderly) that were centred on categories of persons with high visibility or strong symbolic power led to greater participation. In Senegal, the associations and the federations of associations of elderly and retired persons greatly influenced the exemption policy. In 2001, they influenced the recommendation by an interministerial council to create a card that would provide free access to basic social services. In Togo, as was often the case elsewhere in the region, associations of PLHIV were very dynamic in influencing the decision to make ARVs free, as well as in informing their members and monitoring the policy. They worked with their French partners, even going so far as to make demands upon the Global Fund to Fight AIDS, Tuberculosis and Malaria.

#### Patient management and referral (S5)

##### Incomplete exemption policies

Very often, the policies targeted services or categories of persons without considering all the associated needs. For example, in Togo, the pre-therapeutic assessment is not free, even though it is required in order to qualify for free ARV therapy. In Mali, even though ACTs are free at the point of service, patients need to pay for the consultation to obtain them.

##### Partial referral-evacuation systems

Only Burkina Faso included in its policy the funding of transportation between health centres and the district hospital, but it did not reinforce its fleet of ambulances. No country has implemented a system to subsidize transportation between villages and health centres. In Niger, some districts even instituted a new cost-sharing system for referrals in which beneficiaries of the exemption policies are expected to contribute as well (until 2010). Thus, almost none of the countries took advantage of these policies that were focused on financial barriers to also address geographic barriers.

### Actors’ attitudes

Above and beyond the technical and operational aspects discussed above, the actors’ reactions and attitudes are crucial to these policies’ success. It is they who, at the end of the day, make it possible for the policies to be organized and to produce their effects. Yet, despite the primordial importance of these attitudes, the country teams had some difficulty obtaining data on this subject and dealing systematically with it in their knowledge aggregation exercises.

#### Health workers’ motivation and satisfaction (A1)

##### Objectives that were appreciated

In all the countries, the majority of the health personnel approved of the objectives of the user fees exemption policies, which they considered to be fair. Nevertheless, there appeared to be one exception to this general approval. In Senegal, some health professionals seemed to question targeting all the elderly without considering their capacity to pay. But they did not venture to take on the known difficulties of selecting the poorest among this category of persons.

##### Dissatisfaction with the implementation

While the principles underlying these policies appear to be well appreciated, health workers did not hide their dissatisfaction regarding the policies’ implementation. In Burkina Faso, they complained of a lack of medical and technical supplies, while in Senegal and Niger the complaints were regarding significant delays in reimbursement of free services provided to patients. Finally, in most cases, workers were calling for financial bonuses to compensate for increases in their clinical or administrative activities resulting from user fees exemption policies. These financial aspects of bonuses for health workers were not taken into account in any of the policies.

#### Provider–patient relationship (A2)

##### Specific tensions between healthcare providers and patients

Very little information is available on this subject in the country teams’ studies. But overall, it can be seen that the user fees exemption policies sometimes created a *specific tension* between providers and recipients of care, which was less the case in Senegal, where the demands of the elderly were accepted. In other countries, it was not so much increases in activities (which remain *a priori* manageable) as weaknesses in the policies’ implementation that generated such tensions. This was, for example, the case in Mali where there were stock shortages, and in Togo because of the non-comprehensiveness of the exemption, which was limited only to ARVs.

#### Patients’ satisfaction (A3)

##### Overall satisfaction among patients

In all the countries, the population’s overall assessment of the policies appeared positive. The principle of not having to pay at the point of service was thus favourably received.

##### But still some problems

Nevertheless, as with the health workers, the incomplete coverage of services (in Togo, for example) or lack of information on the provisions of the policy (in Senegal and Mali) sometimes produced dissatisfaction among patients. In Burkina Faso, the fact that deliveries were not fully exempted from user fees and that women still had to pay part of the cost also led to some dissatisfactions. Likewise, there appeared to be continuing problems related to the intake and admission process for pregnant women, which reinforced this negative feeling.

#### Patients’ perceptions on health and on financial access to care (A4)

##### Perception that while a financial barrier has been removed, other barriers persist

Everywhere, users perceived the changes introduced by user fees exemption measures in a positive light. However, this positive perception was clearly not absolute, since for some policies the financial barrier was not entirely eliminated. In Burkina Faso, women still have to pay a share of the cost; in Mali, access to free ACTs entails payment for consultations; in Senegal, some drugs for chronic illnesses are not covered. And for nearly all the policies, geographic barriers persist because these policies are almost never concerned about this determinant of access to services.

#### Health workers’ coping strategies (A5)

In several countries the teams discovered certain forms of coping strategies among the health workers or administrative staff when the policies were implemented. These coping strategies were essentially linked to poor functioning of the mechanisms for reimbursement of user fees exemptions.

##### A reorganization of practices

The user fees exemption for ARVs in Togo led to an increase in the number of persons treated, but was organized in a context of a shortage of pharmacists. This made it necessary to create and recruit a new type of intermediate worker, medication dispensers. In addition, physicians had to modify their medical practices because they were dealing with new types of patients in highly sensitive situations requiring careful psychological management. These were people who previously would not have sought care and who did not understand why they had to undergo a pre-treatment work-up, especially since it took a long time for the results to come back. They also did not understand that these tests were obligatory to establish ARV eligibility; they assumed they would automatically receive ARVs, and the idea that they might be refused was inconceivable to them. In Niger, a regional hospital that had not been reimbursed (and therefore had no drugs) asked patients to obtain the drugs in nearby private pharmacies, assuring those pharmacies that it would reimburse them. In primary care centres, health workers wrote prescriptions so that families could go to the private pharmacies knowing what they needed. However, the Ministry of Health subsequently issued directives prohibiting such practices.

##### Service rationing due to lack of reimbursement

In a context in which health centres have fixed costs, and faced with reimbursement delays for free services provided by health workers, some workers in Senegal tried to dissuade elderly patients from using services. For example, some workers gave out appointments for dates in the very distant future, or limited the maximum number of patients seen per day. In Togo, physicians did not immediately apply the free ARV policy.

##### Overcharging or shifting of resources

In Senegal and Burkina Faso, the fact that hospitals combine all revenues into a single account has sometimes led to the funds allocated for user fees exemptions being applied instead to other health centre purposes, putting the application of the policy at risk. But in Senegal, this shifting of funds has also been positive, such as when the resources of a more economically viable service (e.g. maternity) were used to purchase cleaning products for the geriatrics service, an expense not reimbursed by the State. In the same two countries, overcharging practices have also sometimes been observed (real expenses in Senegal, or related to an error in calculating the fixed-rate amount in Burkina) for services that are supposed to be free.

#### Synthesis

Before embarking on the discussion, we present in Table 4 a synthesis of the key results.

**Table 4:** Synthesis of the results of the transversal analysis for the six countries’ knowledge aggregation process

***Implementation Component***	
Management (S1)	Majority of State financing
	Maintenance of centrally organized financing
	Multiplicity of reimbursement methods
	Reimbursement delays and/or stock shortages
	Almost no implementation guides
	Lack of support measures
Communication (S2)	Communication plans that were rarely carried out, funded or renewed
	Health workers who were given general information but not details
	Poorly informed populations
Monitoring / Evaluation / Coordination (S3)	Almost no monitoring and evaluation systems
	Ineffective and poorly funded coordination systems
Community involvement (S4)	Low levels of community involvement
Patient management and referral (S5)	Incomplete exemption policies
	Partial referral-evacuation systems
***Actors’ Attitudes***	
Health workers’ motivation and satisfaction (A1)	Objectives that were appreciated
	Dissatisfaction with the implementation
Provider–patient relationship (A2)	Specific tensions
Patients’ satisfaction (A3)	Overall satisfaction
	But still some problems
Patients’perceptions on health and on financial access to care (A4)	The financial barrier has been removed but other barriers persist
Health workers’ coping strategies (A5)	Reorganization of practices
	Service rationing due to lack of reimbursement
	Overcharging or shifting of resources

Sources: country case studies.

## Discussion

### Methodological strengths and limitations

The main strength of this transversal analysis is that it is not based only on a thorough analysis of the reports produced in each country. In fact, numerous discussions within and between the country work groups, as well as the presentation of the preliminary results of this analysis, also guided the writing of this article. These data confirm what was already available in the scant scientific literature on the subject, which was essentially focused on other African countries [4,9,22]. This therefore confers a certain degree of credibility and external validity on the results presented in this article.

One weakness, which is at the same time a strength of knowledge aggregation processes and of approaches that involve key users in evaluation processes [17], is that much of the data used came from the experience, understanding and discussions of actors responsible for implementing the policies they were invited to study. Of course, conflicts of interest were possible, especially in contexts where applying reflective and critical approaches is rather delicate. However, on one hand, the authors of the case studies did not always maintain a placid discourse (witness the country case studies [14] and the critical data presented in this article) and, on the other hand, since the authors of the present article are fully knowledgeable about the context of these policies’ implementation, they were able to produce this transversal analysis with the critical objectivity required for such an exercise.

### Implementation components and actors’ attitudes revealed by the knowledge aggregation process

As we have said previously, the knowledge aggregation process undertaken in each country was, in itself, a tangible result because “*the process of generating knowledge was the product”*[23]. Program evaluation theorists call this type of approach “process use” to emphasize that these processes’ results begin to be used already during the course of the process [24]. Here we would like to show that these knowledge aggregation exercises also enabled the production of new knowledge that reinforced the current state of knowledge on user fees exemption policies in Africa.

When this study was initiated (2009), there was scant published knowledge on the West African experience with this subject. Also, the literature survey conducted to help the country teams orient their studies provided a status report on public policies and NGO programs [18]. The quantity of knowledge thus available from this survey is presented in the second column of Table 5. The third column of this table shows that the country case studies and the present transversal analysis contribute knowledge on each component of the implementation components and actors’ attitudes. Certainly, this overall vision obscures some particular features. Moreover, we do not have the means to verify whether an improvement in the quantity of available knowledge has been accompanied by an improvement in quality; this remains to be studied. Table 5 confirms that the stakeholders in the national knowledge aggregation exercises were more effective in discussing implementation components, particularly the component related to management (S1), than actors’ attitudes. The reflects, in particular, the fact that these persons did not always have the time, the inclination and the expertise to analyze actors’ attitudes, and that research studies on this topic (which they nevertheless could have used) are still rare [9,25].

**Table 5:** Knowledge available on the policies

	**Knowledge availability**	
	Literature survey*	Knowledge aggregation process**
***Implementation components***		
Management (S1)	+ +	+ + +
Communication (S2)	+ +	+ +
Monitoring / Evaluation / Coordination (S3)	+ +	+ +
Community involvement (S4)	+	+
Management and referral of patients (S5)	+	+ +
***Actors’ attitudes***		
Health workers’ motivation and satisfaction (A1)	+	+ +
Provider–patient relationship (A2)	+	+
Patients’ satisfaction (A3)	+	+
Patients’ perceptions of the effects (A4)		+
Health workers’ coping strategies (A5)	+	+

Note *: produced by the literature survey [18] **: produced by the present transversal analysis.

Given the importance of the implementation processes described in the country teams’ studies, which confirm the scientific literature on this subject [9,22,26], we would like to draw attention in the following paragraphs to some of the shared major problems encountered by most of the countries, before presenting some particularly innovative and therefore potentially inspiring practices.

### Major problems shared by all the countries

The following are elements of implementation that appeared to us to have been fundamental problems in the countries involved in this knowledge aggregation project.

#### Few measures to support the policies

Decisions were often focused only on abolishing or subsidizing user fees without taking into account other determinants of service utilization and the opportunities presented by such abolition to reorganize the healthcare system.

#### Calculation of amounts, reimbursement delays and financial viability

When the countries decided to organize their policies in the form of reimbursements to health centres for services delivered at no charge to patients, they experienced many problems in calculating the amounts to be reimbursed. Often there were no information systems or expertise available. Then, once a system was put in place, the reimbursement methods everywhere were complex, slow and always delayed. While financing was most often included in the national budget, delays in reimbursement were partly due to the fact that the amounts voted into the budgets were not sufficient to meet the demands. In cases of vertical free healthcare policies (e.g. malaria, AIDS) financed by international funding agencies, financial viability was major issue. Likewise, policies not specifically associated with any vertical program in particular appeared to have more difficulty in getting funded than did the others.

#### Public finances organization that was poorly adapted to these changes

The corollary to the preceding problem was that these reimbursement systems were organized in public administrations that remained very centralized and followed public accounting principles that were not appropriate to this task. The New Public Management principles and decentralization have not yet been incorporated into the functioning of administrations. Experiments with using results-based funding approaches are likely to face the same problems in these contexts.

#### Poor traceability of public finances

Most of the country teams had difficulty obtaining numerical data on the budgets actually allocated to these exemption policies. The same was true for the distribution of funding sources for these policies. This problem is clearly related to the preceding two and appears to be even more pronounced for policies not oriented toward any one disease in particular.

#### Lack of reflection on equity and indigence

Most often, the policies targeted broad categories of population without any specific reflection on matters of equity or on subgroups within these populations that were often selected because they were identified as vulnerable (or linked to the MDGs). None of the policies set up activities specifically to promote access to the user fees exemption by the indigent and the poorest. Only Burkina Faso considered this, but never implemented it, even though we demonstrated that the poor benefited more from this policy than did others [27]. It continues to be essential to document the equity impacts of these exemption policies, but at this time, to our knowledge, there is no evidence to show that they do not benefit the worst-off. Equity was therefore the subject of discussion in a workshop organized by the HHA Financial Access to Health Services Community of Practice in September 2012 attended by eight African countries, of which many representatives were stakeholders in the project studied in this article (see: http://www.hha-online.org/hso/marrakesh).

#### No evaluation of the policies

The inadequacy of evaluation measures encountered in the region extends to these exemption policies as well. Thus, it is often difficult to obtain evidential data. Almost no countries have chosen to first implement trials of these exemption measures; instead, most often the exemptions have been organized nation-wide, making it difficult (and sometimes impossible) to evaluate causal attributions for these innovations. As well, we have observed that more funds are available from external agencies for evaluating free healthcare policies targeting specific diseases than for policies that address more general health issues (such as free deliveries).

#### The role of management committees

These exemption policies were organized within the BI cost-recovery context managed by community-based management committees. Despite the known difficulties of real democratic community involvement [28-30], the windows of opportunity opened by these new exemption policies were not seized upon to revive this community-based participation. As with the BI, these committees largely confined themselves to financial management. Likewise, there was no effort to take advantage of the existence of a few health mutuals in the region to strengthen them and make them, when appropriate, vehicles for this exemption policy (by receiving subsidies, for example).

#### Health workers’ bonuses

Health workers definitely have a great deal of power in these policies’ implementation. Yet no country has addressed the issue of health workers’ calls for bonuses whenever an innovation is introduced into the system. At this time, there is no data to show that the (real) increase in their workload is on average insurmountable. However, if this increase is sustained and if these policies are better implemented, then the organization of work, and therefore support measures in terms of human resources and salary bonuses, will need to be addressed.

### Specific features and avenues for research

Beyond these shared difficulties, we also uncovered a certain number of practices that were not previously documented in the literature on user fees abolition policies and that could inspire some researchers to carry out more in-depth studies.

#### Targeting of the elderly (Senegal)

To our knowledge, this is the only country to have tried this experiment of targeting the elderly. Certainly, the political context partly explains this decision, given that Senegal has also instituted user fees exemptions for deliveries and caesareans [21,31].

#### Medication dispensers replacing pharmacists (Togo)

In reflecting on the delegation of tasks, given the increasing numbers of patients receiving ARVs thanks to the exemption, Togo created medication dispensers to compensate for the difficulty of finding enough pharmacists.

#### Administrative directives to maintain the exemption (Niger)

In Niger, in the face of reimbursement delays jeopardizing health centres’ operations and the provision of services to patients, we saw that: i) health workers wrote prescriptions so patients could get what they needed outside, and ii) a direct payment system was set up to co-finance the evacuation of patients. Since these two coping strategies run counter to the exemption, the Ministry issued two directives to prohibit these practices, although the effectiveness of these directives and the extent to which they are routinely applied is not known.

#### Reimbursement of drugs (Senegal)

Instead of reimbursing free services based on a fixed-rate system, the Senegalese authorities experimented with giving drug vouchers to primary care health centres. In these cases, once the drugs were obtained, the vouchers were entered into the routine cost-recovery system and the health centre was be reimbursed.

#### Decentralized focal points (Mali, Niger, Togo)

In these three countries, despite (or because of) a public administration that was still highly centralized, it was decided some time after the exemption measures’ implementation to delegate the policy-monitoring responsibility to persons at decentralized levels of the Ministry of Health.

#### Creation of an independent agency (Benin)

To our knowledge, this was the only experience in the region where the State set up an independent agency to implement the user fees exemption for caesareans. Obviously, this did not resolve all the problems, since there are reimbursement delays and implementation difficulties, but it is an interesting experiment [32].

## Conclusions

This case study analysis of user fees exemption policies for certain services in six West African countries contributes to the construction of a knowledge base that nevertheless remains limited for this African region. These knowledge aggregation exercises undertaken in each country and supported by a team of external researchers, in which policy stakeholders employed both tacit and scientific knowledge, confirmed the importance that must be given to preparation and implementation of these exemption measures. They also show the importance of “community of practice” processes. However, the methodological support and the production of analytical outputs made it possible to extend the process further than traditional discussion workshops, which are indispensable but not sufficient. Over and above the social and political diversity of these countries, the analysis revealed a certain number of points in common, particularly with respect to recurrent implementation challenges [10,33]. Some exploratory avenues of research also began to germinate based on certain practices that emerged from the studies.

The analysis also allows for a prospective reflection on three of the next challenges most likely to confront the countries of that region.

1. Along the lines of Senegal’s current planning, the multiplicity of category-based exemption policies implemented in recent years will compel countries to organize ways of coordinating and harmonizing these measures. Indeed, it will be important to avoid falling into (or accentuating, if it is already the case) the snare of verticality that primary care services promoted over the past 20 years have aimed to counter.

2. In the current wave of social protests in Africa, the place and the role of civil society (NGOs, community management committees, unions, etc.) in these policies remain vague and circumscribed. Like many public policies, those related to user fees exemptions have often been politicized. For these policies to endure, and even develop, beyond the impact of their initial announcement, the involvement of civil society remains a challenge to be considered. This civil society was absent, for example, from the September 2012 HHA community of practice workshop on equity. It should be possible to involve civil society in defining policies and selecting options, as well as in monitoring the policies to ensure they are properly implemented according to plan and public resources are used wisely, as was done recently by an NGO coalition (CROISAD) in Burkina Faso.

3. This sustainability requires that the policies be more deeply anchored socially and that the population’s confidence in the healthcare system be strengthened (which the exemption policies promote), but there clearly also needs to be some assurance that the State will pursue its efforts to consider health a right, and will allocate the public budgets required for universal healthcare access [34]. Therefore, in the current debate around organizing national health insurance systems, incorporating (as in Ghana) these user fees exemption measures will be the next operational challenge.

## Competing interests

The authors of this article initiated the knowledge aggregation project on public policies in the six countries. They supported those responsible for writing the case studies in each country in order to ensure the rigour of their approaches.

## Authors’ contributions

VR and LQ were in charge of the multiple case study designs with the assistance of YK. VR, LQ and YK were in charge of the training and coaching of six country teams in the national case study process (including field visits in the countries). ER did the literature review, developed the analytical framework and assisted VR and LQ in the whole process. VR conducted the transversal data analysis based on the report freely accessible in French at: http://www.vesa-tc.umontreal.ca/pdf/2012/livre_CAPI.pdf. All authors contributed to the interpretation of the results. VR wrote the manuscript with contributions from all authors. All authors had full access to all of the country case studies and take responsibility for the integrity of the data and the accuracy of the data analysis. All author read and approved the final manuscript.

## Pre-publication history

The pre-publication history for this paper can be accessed here:

http://www.biomedcentral.com/1472-6963/12/409/prepub
